# HBsAg and HBeAg in the prediction of a clinical response to peginterferon α-2b therapy in Chinese HBeAg-positive patients

**DOI:** 10.1186/s12985-016-0640-1

**Published:** 2016-10-28

**Authors:** Song Yang, Huichun Xing, Yuming Wang, Jinlin Hou, Duande Luo, Qing Xie, Qin Ning, Hong Ren, Huiguo Ding, Jifang Sheng, Lai Wei, Shijun Chen, Xiaoling Fan, Wenxiang Huang, Chen Pan, Zhiliang Gao, Jiming Zhang, Boping Zhou, Guofeng Chen, Mobin Wan, Hong Tang, Guiqiang Wang, Yuxiu Yang, Dongping Xu, Peiling Dong, Qixin Wang, Jue Wang, Fernando A. Bognar, Daozhen Xu, Jun Cheng

**Affiliations:** 1 Center of Hepatology, Beijing Ditan Hospital, Capital Medical University, No. 8 Jingshun East Street, Chaoyang District, Beijing, 100015 China; 2 Southwest Hospital Affiliated Third Military Medical University, Shapingba, Chongqing 400038 China; 3 Guangzhou Nanfang Hospital, Guangzhou, 510515 China; 4 Xiehe Hospital Affiliated Tongji Medical College of Huazhong University of Science & Technology, Wuhan, 430022 China; 5 Shanghai Ruijin Hospital affilated Shanghai Jiaotong University School of Medicine, Shanghai, 200025 China; 6 Tongji Hospital Affiliated Tongji Medical College of Huazhong University of Science & Technology, Wuhan, 430030 China; 7 The Second Affiliated Hospital, Chongqing Medical University, Chongqing, 400010 China; 8 Beijing You’an Hospital, Capital Medical University, Fengtai District, Beijing, 100069 China; 9 The First Affiliated Hospital of Zhejiang University Medical College, Hangzhou, 310003 China; 10 Peking University People’s Hospital, Beijing, 100044 China; 11 Jinan Infectious Disease Hospital, Jinan, 250021 China; 12 No. 1 Hospital of Chongqing Medical University, Chongqing, 400016 China; 13 Fuzhou Infectious Disease Hospital, Gulou District, Fuzhou, 350025 China; 14 The Third Affiliated Hospital of Sun Yat-sen University, Guangzhou, 510630 China; 15 Huashan Hospital, Fudan University, Shanghai, 200040 China; 16 Shenzhen Donghu Hospital, Shenzhen, 518020 China; 17 Beijing 302 Hospital, Beijing, 100039 China; 18 Shanghai Changhai Hospital, Shanghai, 200433 China; 19 West China Hospital, Sichuan University, Chengdu, 610041 China; 20 Peking University First Hospital, Beijing, 100034 China; 21 Henan Provincial People’s Hospital, Zhengzhou, 450003 China; 22 Merck, Sharp & Dohme, Shanghai, 200040 China; 23 Merck Sharp & Dohme Corp., 600 Corporate Drive, Lebanon, NJ 08833 USA

**Keywords:** Chronic hepatitis B, Hepatitis B surface antigen (HBsAg), Peginterferon alfa-2b, Hepatitis B e antigen (HBeAg), Combined response (CR)

## Abstract

**Background:**

This study aimed to evaluate the predictive values of hepatitis B e antigen (HBeAg) and hepatitis B surface antigen (HBsAg) levels in 171 Chinese patients with chronic hepatitis B who received a 48-week course of pegylated interferon alfa-2b therapy at 1.5 mcg/kg.

**Methods:**

HBsAg, HBeAg, and hepatitis B virus (HBV) DNA levels were measured at baseline and weeks 12, 24, 48, and 72. Clinical responses were defined as a combined response (CR, HBeAg seroconversion [sustained response, SR] combined with HBV DNA level <2,000 IU/mL at week 72). The positive predictive value and negative predictive value were calculated for HBsAg alone and/or combined with HBeAg and HBV DNA at weeks 12 and 24.

**Results:**

Of 171 patients included, 58 (33.9 %) achieved a SR. Of patients who achieved a SR, 33 (56.9 %) achieved a CR. Totally 19.3 % (33/171) patients achieved CR and 80.7 % (138/171) patients did not. Patients with HBsAg <1500 IU/mL at week 12 had a 47.4 % chance of achieving an off-treatment SR and patients with a HBsAg decrease >1.5 logIU/mL at week 12 had a 54.5 % chance. Patients with HBsAg >20,000 IU/mL at weeks 12 and 24 had a 93.8 and 100.0 % chance, respectively, of not achieving a CR. An HBsAg level or changes at weeks 12 and 24, combined with HBeAg or HBV DNA, increased the chance for a SR and CR.

**Conclusions:**

On-treatment HBsAg quantification, alone or in combination with HBeAg or HBV DNA, predicted off-treatment SR and CR after 48 weeks of PEG-IFNα-2b therapy, and thus, may guide clinicians in making a therapeutic decision to continue or terminate the therapy.

## Background

Chronic hepatitis B (CHB) affects more than 240 million people worldwide (http://www.who.int/mediacentre/factsheets/fs204/en/, WHO Fact sheet, updated July 2016), and in China alone, 30 million people are chronically infected with an annual hepatitis B virus (HBV)-related mortality of 300,000 [1, 2]. CHB progresses to liver cirrhosis, liver failure, and even hepatocellular carcinoma [2, 3]. Treatments to suppress HBV and slow the progression of liver inflammation are clinically important.

In general, HBsAg seroconversion (HBsAg loss followed by the appearance of anti-HBs) is the ideal endpoint of antiviral therapy, but it is rarely achieved. Induction of a sustained off-therapy virological and biochemical response in HBeAg-negative patients (either HBeAg-positive cases at baseline with durable anti-HBe seroconversion or HBeAg-negative cases from baseline) is a satisfactory end point, because it has been shown to be associated with improved prognosis [4–9]. Pegylated interferon (PEG-IFN) possesses both antiviral and immunomodulatory effects and is one recommended therapy for CHB patients [7–9]. PEG-IFN can produce a robust off-treatment response in CHB patients [10–13]. However, only one-third of treated patients show a response to PEG-IFN therapy [14]. Thus, it is important to identify patients with a high probability of achieving a satisfactory end point or non-responders as early as possible for optimal clinical management and treatment cost-effectiveness. At present, several virological biomarkers such as HBsAg, HBeAg, and HBV DNA levels are reported to be predictive of the long-term response with PEG-IFN treatment [15–21].

Prediction of off-treatment clinical responses by baseline and on-treatment HBsAg levels as well as other HBV biomarkers has been well established in PEG-IFN–treated patients [20, 22–24]. However, these parameters have negative prediction values (NPVs) as high as 97–100 %, but relatively low positive prediction values (PPVs). Whether combining different biomarkers can improve the PPV has not been well studied. The present study aimed to evaluate the predictive values of on-treatment HBsAg, alone or combination with HBeAg and HBV DNA, for different clinical responses in Chinese HBeAg-positive CHB patients treated with PEG-IFN alfa-2b.

## Methods

### Patients

This study was a post-hoc analysis of P05170, an Asian regional, multicenter, randomized, controlled study (NCT 00536263) evaluating the safety and efficacy of different dosing regimens in treatment-naïve Chinese patients with chronic hepatitis B [25]. Patients were eligible for the P05170 study if they were between 18 and 65 years of age, had CHB for at least 6 months, were positive for HBeAg and HBsAg, had an alanine aminotransferase (ALT) level between 2 and 10 times of upper limit of normal (ULN) range, had HBV DNA levels exceeding 20,000 IU/mL, and had not received IFN therapy within the previous 6 months. Patients were excluded if they were co-infected with other types of hepatitis viruses (A, C, D, or E) or HIV; had decompensated liver disease; had previously received antiviral therapy within 6 months of randomization; or had pre-existing co-morbidities considered unsuitable for enrollment. Patients visited an outpatient hepatology clinic at each participating site for clinical and HBV laboratory examinations every 12 weeks.

For the current study, patients who were initially randomized to a 48-week course of PEG-IFN therapy (1.5 μg/kg weekly) and had completed baseline, end of treatment, and a 24-week follow-up clinical/laboratory assessments (per protocol) were included in the analyses. A PEG-IFN dose of 1.5 μg/kg by 48 weeks was recommended as a standard of care by published guidelines [5, 10]. It is reasonable to set up a prediction profile of HBsAg/HBeAg levels using this PEG-IFN dosing scheme to reflect clinical needs. Therefore, of 220 patients randomized in the P05170 with available data, 171 patients met the criteria for inclusion in the current study. This study was conducted in accordance with the guidelines of the Declaration of Helsinki and the principles of Good Clinical Practice (ICH-E6). Written informed consent according to standards of the local ethics committees was obtained from all patients.

### Laboratory measurement

All blood samples were stored in a central laboratory (LabCorp, Beijing, China) under constant conditions for samples containing hepatitis virus B (−80 °C). HBeAg and HBsAg levels were measured at a central laboratory in serum samples collected at baseline, every 12 weeks during the treatment period, and during the follow-up in the P05170 study. HBsAg levels were quantified using the Elecsys® HBsAg II quantitative assay (electrochemiluminescence immunoassay, Roche Diagnostics, Indianapolis, IN) by Modular Analytics E170 (Roche Diagnostics, (Roche Diagnostics, Switzerland) with a range of 5–52,000 IU/ml for up to a 400-fold-diluted sample. HBeAg levels were quantified using the Elecsys® HBeAg II quantitative assay (Modular Analytics E170 (Roche Diagnostics, (Roche Diagnostics, Switzerland) with a quantification lower limit of 5 IU/mL. HBV DNA quantification was performed using Taqman-based polymerase chain reaction (PCR) assays (COBAS® AmpliPrep/COBAS® TaqMan48, Roche Molecular Systems Inc.) with a lower limit of 6 IU/mL. HBV genotype was assessed using the INNO-LiPA line probe assay (Innogenetics, Ghent, Belgium). ALT was measured locally in accordance with standard laboratory procedures in P05170 and was retrieved and presented as a multiple of the ULN to minimize inter-laboratory assay bias.

### Outcomes

In this study, sustained response (SR) was defined as HBe seroconversion (HBeAg loss and positivity for anti-HBeAg) at 24 weeks after the end of a 48-week treatment. Combined response (CR) was defined as a combination of SR and HBV DNA < 2000 IU/ml at 24 weeks after the end of a 48-week treatment. The cohort was divided and presented by responder and non-responder groups according to achievement of a CR.

### Statistical analysis

All analyses were based on the responder versus non-responder groups. Within- and between-group statistical comparisons for HBsAg, HBeAg, and HBV DNA levels during the treatment and at the end of study were achieved by using mixed model repeated measures with no adjustment for covariates. Predictive accuracy was analyzed and presented as PPV and NPV for SR and CR, by setting cut-offs for HBsAg levels, alone or in combination with HBeAg or the HBV DNA levels. The area under the receiver operating characteristic (ROC) curve (AUC) was assessed in terms of the predictive strength of three different on-treatment parameters. The patient’s baseline and on-treatment characteristics were grouped and compared by CR.

Finally, logistic regression models (univariate and multivariate analyses) were constructed to explore the factors that were associated with the clinical response as expressed by CR. All presented baseline and on-treatment characteristics (on-treatment continuous variables of HBsAg, HBeAg, and HBV DNA were not included for the consideration of multicollinearity in the model) were included in the univariate model, and those with a *p*-value <0.1 were selected to enter into multivariate model, where variables with *p* < 0.1 were retained by a hierarchical forward with switching method.

All statistical analyses were performed using NCSS 10 software (NCSS, LLC, Kaysville, UT). All statistical tests were two-sided and performed at the 0.05 level of significance unless otherwise specified.

## Results

### Patient characteristics and clinical response rate

The off-treatment clinical response rates and the selected baseline and on-treatment characteristics are summarized in Table 1. Patients who achieved a CR had significantly lower baseline HBsAg levels (*p* = 0.01) and a shorter duration of HBV exposure, but higher baseline ALT levels as presented by multiples of ULN (*p* = 0.05). Patients who achieved a CR had significantly lower HBsAg, HBeAg, and HBV DNA levels (all *p* values <0.05), compared with those who did not, at on-treatment weeks 12 and 24. Similarly, proportions of patients who had a significant decrease in HBsAg, HBeAg (>1.5 logIU/mL), or HBV DNA (>2.0 logIU/mL) were significantly higher in the groups of patients with a CR at weeks 12 and 24 (all *p* values <0.05).

**Table 1:** Baseline and on-treatment characteristics of the study cohort

Characteristics	Total	CR (+)	CR (−)	*p* value
No. of patients	171	33 (19.3)	138 (80.7)	
Age (years)	28.5 (7.9)	26.7 (7.6)	28.9 (7.9)	0.09
Gender (male)	130/171 (76.0)	24/33 (72.7)	106/138 (76.8)	0.62
BMI	22.2 (3.2)	22.0 (2.9)	22.2 (3.3)	0.71
Years of HBV Exposure	11.2 (9.3)	7.8 (5.5)	12.0 (9.8)	0.02
HBV Genotype				
B	77	14 (42.4)	63 (45.7)	0.74
C	94	19 (57.6)	75 (54.3)	
ALT (*ULN)	4.0 (1.9)	4.6 (2.3)	3.8 (1.8)	0.05
ALT > 5*ULN	35/171 (20.5)	10/33 (30.3)	25/138 (18.1)	0.12
HBsAg (Log10 IU/mL)	4.2 (0.6)	4.0 (0.6)	4.3 (0.6)	0.01
HBeAg (Log10 IU/mL)	2.6 (0.6)	2.4 (0.6)	2.6 (0.6)	0.09
HBV DNA (Log10 IU/mL)	7.8 (0.8)	7.7 (0.7)	7.8 (0.8)	0.44
On-treatment Quantifications, week 12				
HBsAg decrease > 1.5Log	12/171 (7.0)	5/33 (15.2)	7/138 (5.1)	0.04
HBeAg decrease > 1.5Log	46/171 (26.9)	15/33 (45.5)	31/138 (22.5)	0.01
HBV DNA decrease > 2.0Log	51/171 (29.8)	18/33 (54.5)	33/138 (23.9)	<0.01
On-treatment Quantifications, week 24				
HBsAg decrease > 1.5Log	30/171 (17.5)	10/33 (30.3)	20/138 (14.5)	0.03
HBeAg decrease > 1.5Log	69/171 (40.4)	23/33 (69.7)	46/138 (33.3)	<0.01
HBV DNA decrease > 2.0Log	77/171 (45.0)	24/33 (72.7)	53/138 (38.4)	<0.01

Data are presented as Mean (SD) or n (%)

Combined response (CR) was defined as HBeAg seroconversion combined with HBV DNA level <2,000 IU/mL at 24 weeks after the end of treatment (week 72)

### Changes in on-treatment HBV DNA and HBV serological markers

**Fig. 1:**
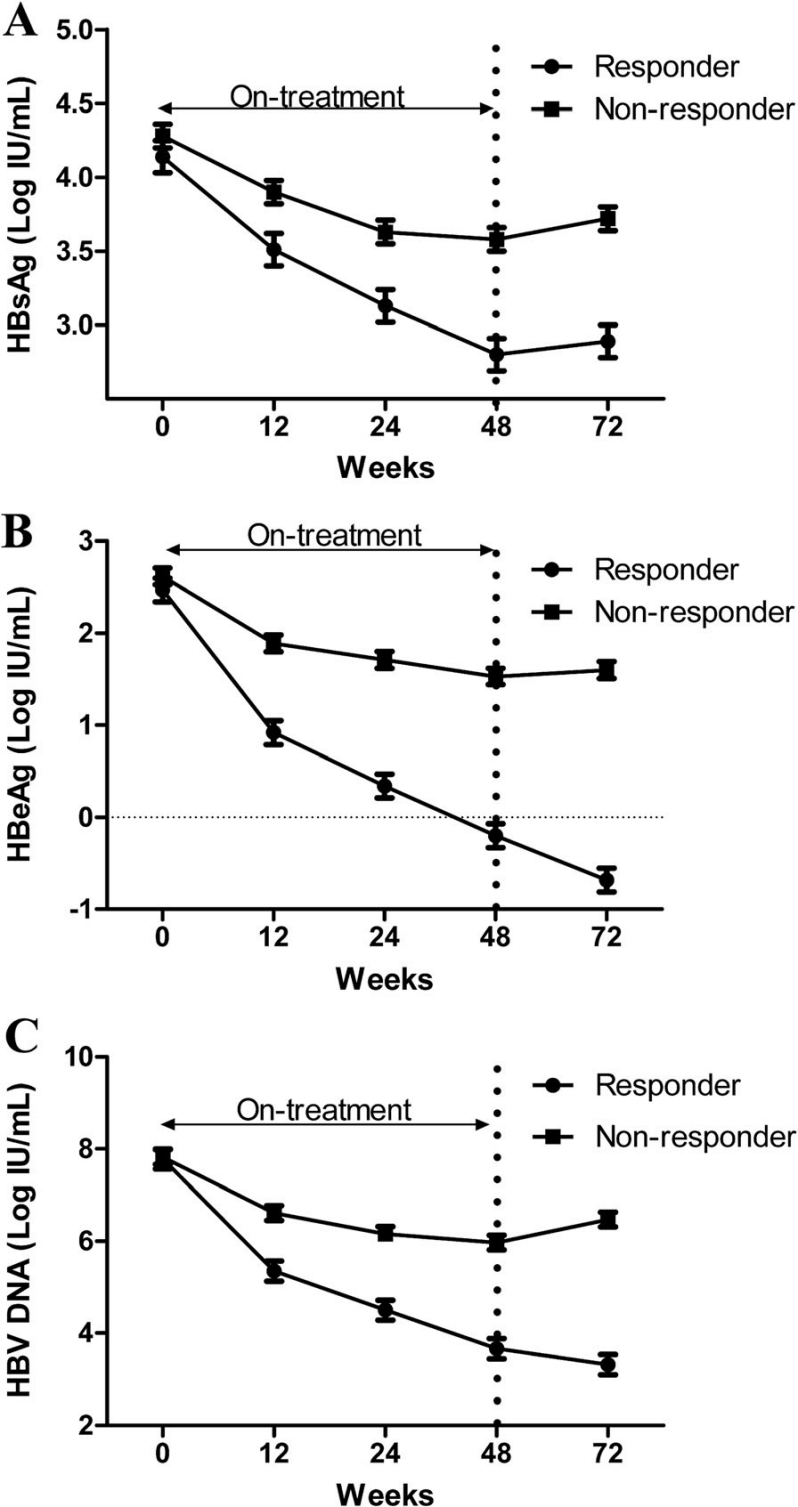
Mean changes (standard error) in HBsAg (**a**), HBeAg (**b**), and HBV DNA (**c**) levels from baseline according to HBe seroconversion response in HBeAg-positive CHB patients receiving PEG-IFN treatment. *All *p*-values <0.01 for between-group comparisons at post-baseline time points weeks 12, 24, 48, and 72. *p*-values <0.01 for within-group comparisons between week 72 and baseline or week 48 for responders who achieved HBe seroconversion

Changes in the quantified levels of HBV biomarkers were analyzed and grouped by patients who achieved a SR versus those who did not (Fig. 1). Overall, patients who achieved a SR had significantly lower HBsAg levels during the treatment and follow-up period than those who did not (all *p* values < 0.01). In patients who had a SR, the HBsAg levels declined significantly during the treatment period and the follow-up, compared with that at baseline (all *p* values <0.01) and slightly rebounded at follow-up week 72 compared with that at the end of treatment (*p* = 0.33). On the contrary, in patients who did not achieve a SR, HBsAg levels at week 72 were significantly higher than those at week 48 (*p* = 0.02).

### Prediction of CR by HBV DNA and HBV serological biomarkers

**Fig. 2:**
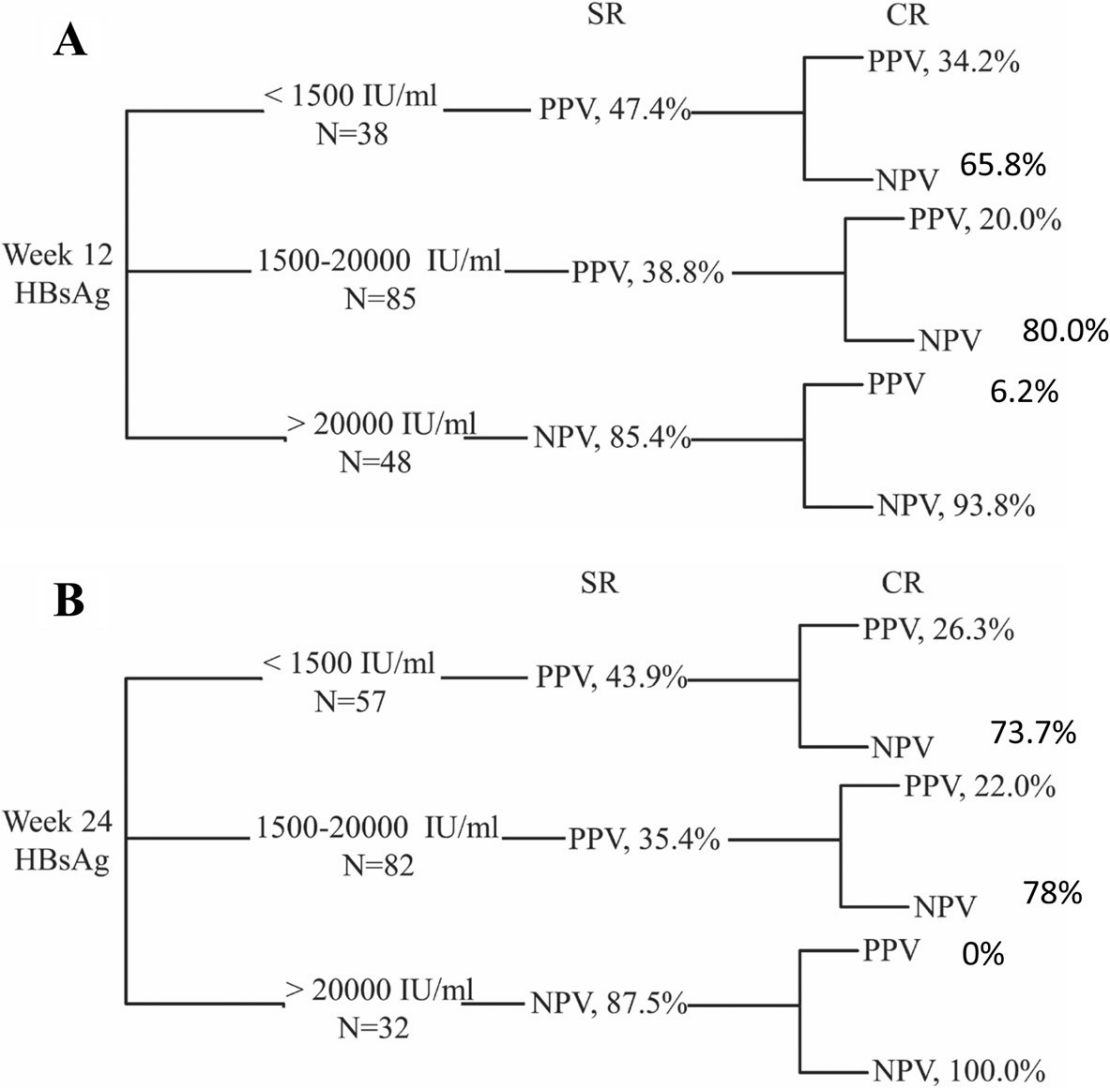
Flow chart for predictive values of on-treatment HBsAg at weeks 12 (**a**) and 24 (**b**) for SR and CR at week 72. Three groups were assigned according to HBsAg levels (<1500, 1500–20000, and >20000 IU/ml), and the respective PPV or NPV to SR and CR are presented

The predictive values of on-treatment HBV biomarker levels by cut-offs in absolute values and changes at weeks 12 and 24 for a CR are shown in Table 2. The PPVs and NPVs of HBsAg quantifications by selected cut-offs (absolute values) for the prediction of an SR and CR at weeks 12 and 24 are shown in Fig. 2. The probability of achieving an off-treatment SR (PPV) was 47.4 % in patients who had HBsAg less than 1500 IU/mL and 54.5 % in patients who had an HBsAg decrease >1.5 logIU/mL at week 12. The PPV for a SR was 54.5 % in patients who had an HBsAg decrease >1.5 logIU/mL at week 12. In patients who had an HBsAg >20,000 IU/mL at week 12, the likelihood of a treatment failure to a CR was 93.8 % (NPV). The NPV for a CR was 100.0 % in patients treated for 24 weeks but who maintained HBsAg levels >20,000 IU/mL (ROC AUC: 0.70, 0.71 at weeks 12 and 24, *p* values < 0.01, data not shown).

**Table 2:** Prediction of CR by HBV DNA and serological biomarkers

		CR (+)	CR (−)	PPV	NPV
HBsAg	Week 12				
	Up to 1500 IU/mL	13	25	34.2	-
	1500 - 20000 IU/mL	17	68	20.0	-
	Over 20000 IU/mL	3	45	-	93.8
	Week 12 Change				
	Up to −1.5 log IU/mL	5	6	45.5	-
	− 1.5 ~ −0.5 log IU/mL	14	37	27.5	-
	Over −0.5 log IU/mL	14	95	-	87.2
	Week 24				
	Up to 1500 IU/mL	15	42	26.3	-
	1500 - 20000 IU/mL	18	64	22.0	-
	Over 20000 IU/mL	0	32	-	100.0
	Week 24 Change				
	Up to −1.5 log IU/mL	10	20	33.3	-
	− 1.5 ~ −0.5 log IU/mL	11	44	20.0	-
	Over −0.5 log IU/mL	12	74	-	86.0
HBeAg	Week 12				
	Up to 10 IU/mL	19	37	33.9	-
	10 - 100 IU/mL	5	31	13.9	-
	Over 100 IU/mL	9	70	-	88.6
	Week 12 Change				
	Up to −1.5 log IU/mL	15	31	32.6	-
	− 1.5 ~ −0.5 log IU/mL	11	34	24.4	-
	Over −0.5 log IU/mL	7	73	-	91.3
	Week 24				
	Up to 10 IU/mL	24	53	31.2	-
	10 - 100 IU/mL	4	24	14.3	-
	Over 100 IU/mL	5	61	-	92.4
	Week 24 Change				
	Up to −1.5 log IU/mL	23	46	33.3	-
	− 1.5 ~ −0.5 log IU/mL	8	29	21.6	-
	Over −0.5 log IU/mL	2	63	-	96.9
HBV DNA	Week 12				
	Up to 20000 IU/mL	12	16	42.9	-
	Over 20000 IU/mL	21	122	-	85.3
	Week 12 Change				
	Up to −2.0 log IU/mL	18	33	35.3	-
	Over −2.0 log IU/mL	15	105	-	87.5
	Week 24				
	Up to 20000 IU/mL	19	32	37.3	-
	Over 20000 IU/mL	14	106	-	88.3
	Week 24 Change				
	Up to −2.0 log IU/mL	24	53	31.2	-
	Over −2.0 log IU/mL	9	85	-	90.4

HBeAg quantification gave a higher PPV for a SR at weeks 12 and 24 than HBsAg (57.1 and 60.9 % vs 47.4 and 54.5 %, respectively). The PPV for a SR was 55.8 and 63.8 % in patients who had an HBeAg decrease >1.5 logIU/mL at weeks 12 and 24, respectively. The NPV for a CR was 96.9 % in patients whose HBeAg decline was <0.5 logIU/mL at week 24. Similarly, trends in PPV analysis were seen in HBV DNA quantification.

**Fig. 3:**
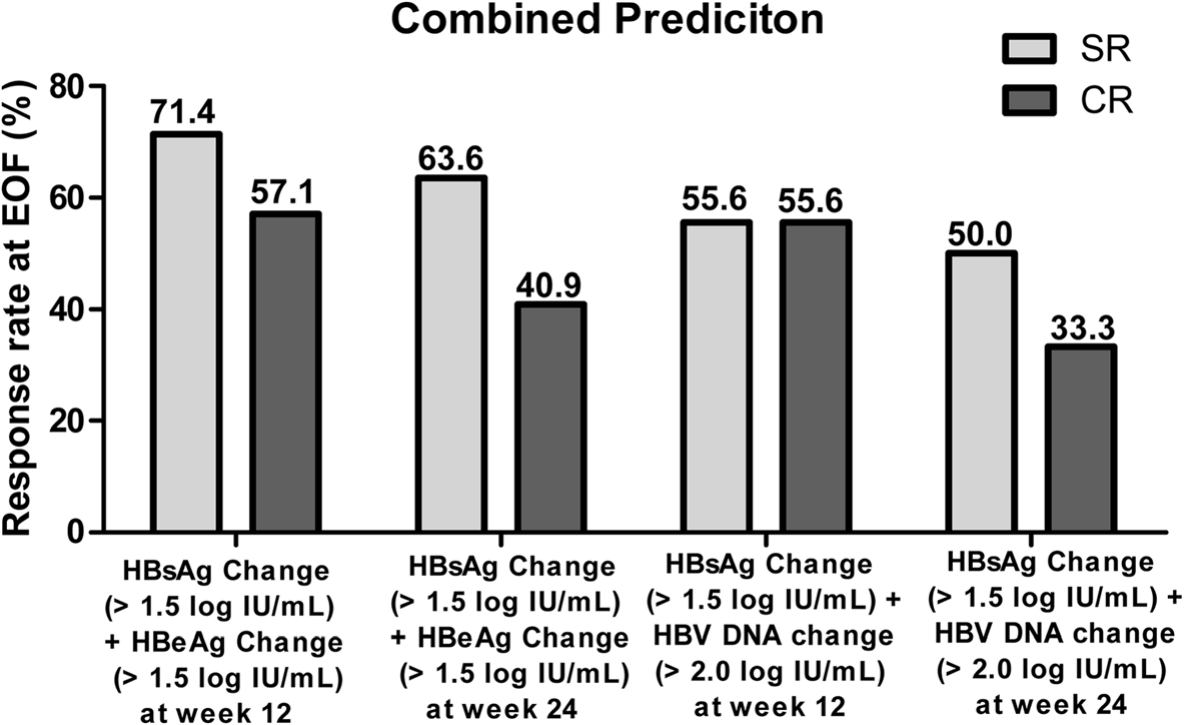
Combined predictive value of changes of HbsAg, HBeAg, and HBV DNA at weeks 12 and 24 for SR and CR at week 72 (end of follow-up, EOF). SR, sustained response; CR, combined response

The predictive accuracy of HBsAg quantification was further explored by adding HBeAg as a combination prediction scenario. The HBsAg decrease by a certain amount (>1.5 logIU/mL) combined with HBeAg (>1.5 logIU/mL), at week 12 and 24, had an increased PPV for a SR [71.4 (5/7) and 63.6 % (14/22) respectively, Fig. 3]; an HBsAg decrease >1.5 logIU/mL at week 12, combined with HBeAg >1.5 logIU/mL or HBV DNA decrease >2.0 logIU/mL at week 12, had an increased PPV for an off-treatment CR [57.1 (4/7) and 55.6 % (5/9) respectively, Fig. 3]. When the HBsAg was decreased >1.5 logIU/mL along with an HBeAg >1.5 logIU/mL or an HBV DNA decrease >2.0 logIU/mL, at week 24, the PPVs for a CR were 40.9 (9/22) and 33.3 % (8/24), respectively (Fig. 3).

### Associated factors for predicting combined response

Exploratory analyses for identifying factors associated with a CR were performed (Table 3). Univariate analysis showed that the mean ALT at baseline (×ULN), mean HBsAg at baseline (logIU/mL), mean HBeAg at baseline (log IU/mL), on-treatment HBsAg decrease >1.5log, HBeAg decrease >1.5log, and HBV DNA decrease >2.0log at weeks 12 and 24 differed significantly between responders and non-responders groups (all *p*-values < 0.1), and therefore, these variables were entered into a multivariate logistic regression model to evaluate their predictive strength for a CR. Multivariate analysis suggested that the baseline HBsAg level and a HBeAg decrease >1.5log at week 24 were significantly associated with a CR (both *p-*values <0.05).

**Table 3:** Univariate and multivariate modeling of baseline and selected on-treatment characteristics

Characteristics	CR (+)			
	Univariate		Multivariate	
	OR (95 % CI)	*p* value	OR (95 % CI)	*p* value
Age	0.96 (0.91, 1.01)	0.14		
Gender (Male)	0.81 (0.34, 1.91)	0.62		
BMI	0.98 (0.87, 1.10)	0.72		
HBV Genotype (B)	0.88 (0.41, 1.89)	0.74		
ALT (*ULN)	1.19 (1.00, 1.43)	0.05		
ALT > 5*ULN	1.97 (0.83, 4.64)	0.12		
HBsAg (Log_10_ IU/mL)	0.41 (0.22, 0.79)	0.01	0.38 (0.19, 0.79)	0.01
HBeAg (Log_10_ IU/mL)	0.61 (0.34, 1.08)	0.09		
HBV DNA (Log_10_ IU/mL)	0.83 (0.51, 1.33)	0.43		
On-treatment Quantifications, week 12				
HBsAg decrease > 1.5Log	3.34 (0.99, 11.30)	0.05	3.36 (0.90, 12.56)	0.07
HBeAg decrease > 1.5Log	2.88 (1.30, 6.36)	0.01		
HBV DNA decrease > 2.0Log	3.82 (1.73, 8.40)	<0.01		
On-treatment Quantifications, week 24				
HBsAg decrease > 1.5Log	2.57 (1.06, 6.19)	0.04		
HBeAg decrease > 1.5Log	4.60 (2.02 10.47)	<0.01	4.02 (1.72, 9.39)	<0.01
HBV DNA decrease > 2.0Log	4.28 (1.85, 9.90)	<0.01		

All baseline and selected on-treatment characteristics were analyzed for CR (*N* = 171) by univariate analysis and were all entered into multivariate model where variables with *p* < 0.1 were retained by Hierarchical Forward with Switching Method

## Discussion

PEG IFN is one of the first-line treatment options for HBeAg positive CHB. Using a randomized controlled trial cohort with CHB treated with a dosing scheme of 1.5 μg/kg PEG-IFN alfa-2b weekly for 48 weeks, the predictive profiles of HBsAg alone and combined with HBeAg and HBV DNA levels for the clinical response were investigated. The response rates reported in this paper were consistent with those reported previously and comparable to those achieved with other pegylated interferon therapies including PEG IFN 2a [24, 26, 27]. The PPVs were relatively lower for the prediction of CR in HBeAg-positive CHB patients treated by PEG-IFN. In combination with HBeAg or HBV DNA with selected cut-offs, HBsAg at weeks 12 or 24 had increased PPVs for predicting an off-treatment response. Moreover, HBsAg with a selected cut-off (>20,000 IU/mL) exhibited a high NPV for CR at weeks 12 and 24.

Currently, PEG IFN is the only treatment choice for HBeAg-positive CHB patients to achieve a sustained off-treatment response. However, the response rate was not satisfactory, with only 30 % of patients achieving a SR, and the rate was even lower for a CR [14]. Considering the low response rate and potential cost and safety issues, it would be clinically beneficial to identify patients who are likely (or unlikely) to develop a desired clinical outcome as early as possible. In the present study, HBsAg, HBeAg, and HBV DNA levels in the clinical responders decreased significantly during treatment and by 24 weeks after the end of treatment; whereas non-responders had a moderate decline at the beginning of treatment and experienced significant rebounding thereafter. These preliminary data were consistent with previous findings [19, 20, 24] and increase the potential for discovering how to individualize PEG-IFN alfa-2b-based therapy according to longitudinal on-treatment HBsAg levels. Recent studies have shown that serum levels of HBsAg are associated with the levels of intrahepatic covalently closed circular DNA (cccDNA), and thus, the HBsAg level has been considered a marker for the prediction of a clinical response [28, 29]. The predictive value of the HBsAg level for a clinical response to PEG-IFN therapies has been validated in CHB patients [30–32]. In the present study, we found HBsAg quantification alone at week 12 or 24 did not produce statistically convincing PPVs for an off-treatment clinical response. A high HBsAg level >20,000 IU/mL at weeks 12 and 24 predicted 93.8 and 100.0 % likelihoods of not achieving a CR. This suggested a stopping rule to discontinue PEG-IFN treatment for patients who have a high absolute value of HBsAg at week 12 or 24. These data were consistent with those of previous studies on PEG-IFN [24, 30, 31].

On-treatment HBeAg quantification or decline also showed a high prediction value for a SR with PEG-IFN treatment. Freid et al. reported that patients with high levels of HBeAg (>100 PEIU/mL) at week 24 had a 96 % probability of no response [21]. Ji et al. reported that 87.5 % of patients with an HBeAg decline >2 log achieved undetectable HBV DNA levels and 75.0 % who had a HBeAg decline >2log achieved HBeAg seroconversion [33]. The HBV DNA level is always predictive of a SR during CHB treatment. Gheorghiţa et al. reported that an on-treatment HBV-DNA reduction of >2 log10 IU/ml with any decrease in the HBsAg level at week 12 has a PPV of 80 % for SR [19]. For practical consideration, the predictive roles of HBeAg and HBV DNA might be used for an adjuvant HBsAg. For patients with low-to-moderate HBsAg readings at week 12 or 24, a clinical decision in terms of treatment continuation may be strengthened by measuring HBsAg in combination with HBeAg or HBV DNA. A combination of HBsAg and HBeAg or HBV DNA at weeks 12 and 24 had generally better PPVs for CR in comparison with HBsAg alone. In particular, a combination prediction scenario did not simultaneously increase NPVs. Considering NPVs according to HBsAg alone, this algorithm gives clinicians clear-cut information for making a decision regarding early treatment discontinuation but suggests more careful evaluations are needed for maintenance of PEG-IFN antiviral treatment when HBsAg levels in the early on-treatment phase are low-to-moderate. However, these data must be interpreted with caution due to the relatively small sample size in analysis of PPVs for combination predictions and the results were preliminary per se.

This post-hoc analysis is among the limited number of studies analyzing and reporting predictive profiles of HBsAg quantification alone and in combination with HBeAg or HBV DNA to identify off-treatment clinical responses in patients treated by PEG-IFN alfa-2b. The analyzed patient cohort can represent the disease population in China, where HBV prevalence is the highest despite the effective use of HBV vaccines. However, several limitations existed. The analyzed patients were not part of a large sample size, which may weaken the statistical interpretation of PPVs and NPVs for clinical practice. The study results for HBsAg in combination with other markers, are preliminary and require verification in patients treated with PEG-IFN alfa-2b. We identified clinical and laboratory factors that were significantly associated with an off-treatment clinical response by constructing a multivariate analysis model. However, due to the lack of internal or external validation, the results must be considered solely preliminary.

## Conclusion

In conclusion, quantified on-treatment HBsAg was a strong predictor for an off-treatment CR in Chinese HBeAg positive CHB patients who had received 48 weeks of PEG-IFN alfa-2b treatment. On-treatment HBsAg quantifications, in combination with HBeAg or HBV DNA, predicted an off-treatment SR and CR after 48 weeks of PEG-IFNα-2b therapy. HBsAg quantification as early as 12 weeks, in combination with HBeAg or HBV DNA, predicted an off-treatment CR after 48 weeks of treatment. These on-treatment predictors may help clinicians decide whether to continue PEG-IFN alfa-2b-based therapy or switch to an alternative therapy in Chinese patients.

## Abbreviations

ALT: Alanine aminotransferase
cccDNA: Covalently closed circular DNA
CHB: Chronic hepatitis B
CI: Confidence interval
HBeAg: Hepatitis B e antigen
HBsAg: Hepatitis B surface antigen
HBV: Hepatitis B virus
NPV: Negative predictive value
PEG-IFN: Pegylated interferon
PPV: Positive predictive value
ROC AUC: Area under the receiver operating characteristic curve
SD: Standard deviation
SR: Sustained response
ULN: Upper limit of normal
